# Outcome of segmental prosthesis reconstruction for diaphyseal bone tumors: a multi-center retrospective study

**DOI:** 10.1186/s12885-019-5865-0

**Published:** 2019-06-28

**Authors:** Kai Zheng, Xiu-chun Yu, Yong-cheng Hu, Zeng-wu Shao, Ming Xu, Bai-chuan Wang, Feng Wang

**Affiliations:** 1 Department of Orthopedics, The 960th Hospital of the PLA Joint Logistice Support Force (previous name: General Hospital of Jinan Military Command), No. 25 Shifan Road, Jinan, 250031 China; 2 Department of Orthopedics, Tianjin Hospital, Tianjin, China; 3 Department of Orthopedics, Union Hospital, Tongji Medical College, Huazhong University of Science and Technology, Wuhan, China

**Keywords:** Segmental prostheses, Diaphysis tumors, Diaphyseal defects, Bone reconstruction, Retrospective study, Pathological fractures, Assistant plate

## Abstract

**Background:**

The optimal reconstructive method after diaphyseal malignant bone tumor resection remains controversial. This multicenter clinical study was designed to investigate the clinical value and complications of segmental prosthesis in the repair of diaphyseal defects.

**Methods:**

We present 49 patients from three clinical centers treated with wide resection for primary or metastatic bone tumors involving the diaphysis of the femur, tibia, humerus, or ulna, followed by reconstruction using a modular intramedullary segmental prosthesis.

**Results:**

Enrolled patients included 23 men and 26 women with a mean age of 63.3 years. Of these, seven patients had primary bone tumors and 42 patients had metastatic lesions. At the mean follow-up of 13.7 months, 17 patients were alive, 31 patients were deceased due to tumor progression, and one patient was dead of another reason. There were eight nononcologic complications (two with radial nerve injury, three with delayed incision healing, two with aseptic loosening in the proximal humerus prosthetic stem and one with structural failure) and three oncologic complications (three with primary tumor recurrence) among all patients. The incidence of complications in primary tumor patients (4/7, 57.1%) was higher than that in patients with metastatic tumors (7/42, 16.7%) (*p* = 0.036). Aseptic loosening and mechanical complications were not common for patients with primary tumors, although the reconstruction length difference was statistically significant (*p* = 0.023). No statistically significant differences were observed in limb function, while the mean musculoskeletal tumor society score was 21.2 in femora, 19.6 in humeri, and 17.8 in tibiae (*p* = 0.134).

**Conclusions:**

Segmental prostheses represent an optional method for the reconstruction of diaphyseal defects in patients with limited life expectancy. Segmental prostheses in the humerus experienced more complications than those used to treat lesions in the femur.

## Background

The reconstruction of diaphyseal defects following malignant tumor resection has long been a challenge. However, the optimal choice of reconstructive method remains controversial [1]. Alternative reconstruction methods include autografts [2, 3], allografts [4, 5], bone transport [6], inactivated bone replantation [7, 8], and segmental prostheses [9–12]. Autografts with fibula or ilium have good bone tissue compatibility, playing an important role in diaphyseal reconstruction, but are limited by autologous bone sources and cause bone defect at the source [1, 3]. Allograft reconstruction can be used in large diaphyseal defects with good matching, but its disadvantages include the risk of disease transfer to the host, allograft rejection, long recovery, nonunion, allograft fracture, and early infection [1, 4]. Bone transport can achieve good biological reconstruction after successful operations. However, a long period of transport time and limb bearing limitation, external fixation pin tract infection, and skin and soft tissue cutting injuries are common complications [6]. Inactivated-bone replantation has good shape matching, but the disadvantages include bone strength decrease and the risk of tumor recurrence [7, 8]. The advantages of segmental prosthesis reconstruction include early mobilization, simple operation, short hospital stay and the ability to tolerate chemotherapy and radiotherapy after incision healing. However, mechanical failure and aseptic loosening of the prosthesis are important concerns [9, 13, 14]. Compared to other reconstruction methods, the available literature on segmental prostheses is scarce and the clinical effects of segmental prostheses are not clear.

This study was performed to better understand the outcomes and complications of segmental prosthesis reconstruction performed after diaphyseal tumor resection.

## Methods

### Patients

We retrospectively studied the medical records of patients treated between 2010 and 2017 at three musculoskeletal oncology centers in China. Inclusion criteria were patients with segmental bone loss from primary malignant or metastatic tumor resection with sparing of the joint, deemed unsuitable for biologic reconstruction, and who were surgically treated with a modular intercalary segmental prosthesis in the diaphysis of the ulna, humerus, tibia, or femur.

We described the surgical indications and patient prognosis, focusing on postoperative limb function, causes of surgical complications, and treatments reducing prosthesis failure. All patients were included at the postoperative evaluation until death or the latest examination for the purpose of this study (Table 1). Adjuvant treatments, including chemotherapy, radiotherapy and targeted therapy, were administered in some patients according to the pathological type of their tumor.

**Table 1:** presents data of 49 patients; it is a case series study

No.	Age range (years)	Location	Diagnosis	Pathological fracture (yes/no)	Resection length (mm)	Plate used (yes/no)	Followup (months)	Complications	MSTS score
1	> 80	Ulna	Undifferentiated sarcoma	Yes	60	No	38	None	28
2	41–50	Humerus	Prostate cancer	Yes	80	Yes	16	None	21
3	61–70	Humerus	Rectal cancer	Yes	90	Yes	6	Radial nerve injuries	15
4	61–70	Humerus	Lung cancer	No	90	No	10	Aseptic loosening loosening 4 mm	20
5	71–80	Humerus	Multiple myeseloma	Yes	90	Yes	3	None	18
6	51–60	Humerus	Lung cancer	Yes	80	Yes	3	None	17
7	51–60	Humerus	Lung cancer	No	80	No	8	None	19
8	> 80	Humerus	Liver cancer	Yes	80	No	4	None	16
9	61–70	Humerus	Breast cancer	Yes	60	No	17	None	23
10	51–60	Humerus	Lung cancer	No	80	No	13	None	22
11	61–70	Humerus	Multiple myeseloma	Yes	85	No	26	Aseptic loosening	25
12	71–80	Humerus	Lung cancer	No	75	No	22	None	22
13	41–50	Humerus	Prostate cancer	Yes	80	Yes	12	None	22
14	61–70	Humerus	Rectal cancer	Yes	90	No	6	Radial nerve injuries	15
15	51–60	Femur	Lung cancer	Yes	80	No	4	None	22
16	71–80	Femur	Rectal cancer	Yes	100	No	3	None	19
17	51–60	Femur	Liver cancer	Yes	110	No	4	None	19
18	31–40	Femur	Osteosarcoma	Yes	110	No	25	Tumor recurrence	16
19	51–60	Femur	Breast cancer	Yes	160	Yes	36	None	27
20	71–80	Femur	Rectal cancer	Yes	90	No	8	None	22
21	61–70	Femur	Renal cancer	Yes	80	No	9	None	24
22	61–70	Femur	Lung cancer	Yes	120	No	10	None	26
23	51–60	Femur	Renal cancer	Yes	80	No	14	None	22
24	61–70	Femur	Breast cancer	Yes	80	Yes	7	None	20
25	61–70	Femur	Liver cancer	Yes	80	No	4	None	19
26	61–70	Femur	Lung cancer	Yes	100	No	9	None	21
27	61–70	Femur	Lung cancer	Yes	80	No	3	Angulation	21
28	71–80	Femur	Multiple myeseloma	Yes	120	No	10	None	25
29	71–80	Femur	Unknown source cancer	Yes	70	Yes	3	Incision delayesed healing	15
30	71–80	Femur	Non-Hodgkin lyesmphoma	Yes	150	Yes	10	None	19
31	71–80	Femur	Lung cancer	Yes	90	Yes	8	None	16
32	51–60	Femur	Lung cancer	No	80	No	12	None	22
33	61–70	Femur	Lung cancer	No	100	No	7	None	18
34	51–60	Femur	Lung cancer	No	120	No	15	None	27
35	> 80	Femur	Renal cancer	Yes	80	No	79	None	28
36	71–80	Femur	Renal cancer	Yes	100	No	38	None	27
37	51–60	Femur	Breast cancer	Yes	60	No	3	None	19
38	41–50	Femur	Lung cancer	No	120	No	14	None	24
39	10–20	Femur	Osteosarcoma	Yes	190	No	3	None	18
40	71–80	Femur	Breast cancer	Yes	60	No	6	None	19
41	71–80	Femur	Renal cancer	Yes	80	No	3	None	16
42	51–60	Femur	Lung cancer	Yes	80	No	4	None	17
43	71–80	Femur	Unknown source cancer	Yes	70	Yes	23	Incision delayesed healing	20
44	71–80	Femur	Non-Hodgkin lyesmphoma	Yes	80	No	46	None	27
45	41–50	Tibia	Langerhans cell sarcoma	No	100	No	21	Tumor recurrence	10
46	61–70	Tibia	Undifferentiated sarcoma	No	100	No	13	Incision delayesed healing	18
47	61–70	Tibia	Unknown source cancer	Yes	60	Yes	21	None	22
48	41–50	Tibia	Soft tissue sarcoma	No	100	No	7	Tumor recurrence	20
49	51–60	Tibia	Osteosarcoma	No	130	No	7	None	19

Preoperative X-ray, CT scan, MR imaging and ECT for all bones were performed. The tumor pathological types were confirmed by pathological examination of tumor biopsies or resected specimens. Patients with primary diaphyseal tumors without pathological fracture were considered for segmental prosthesis reconstruction if their bone strength evaluation score was higher than 8 points [15]. Patients with metastatic diaphyseal tumors without pathological fracture accepted segmental prosthesis reconstruction if the obtained Mirel’s score [16] was more than 9 points. All patients with pathological diaphyseal fractures accepted segmental prosthesis reconstruction if the patient’s physical condition could tolerate surgical treatment.

**Fig. 1:**
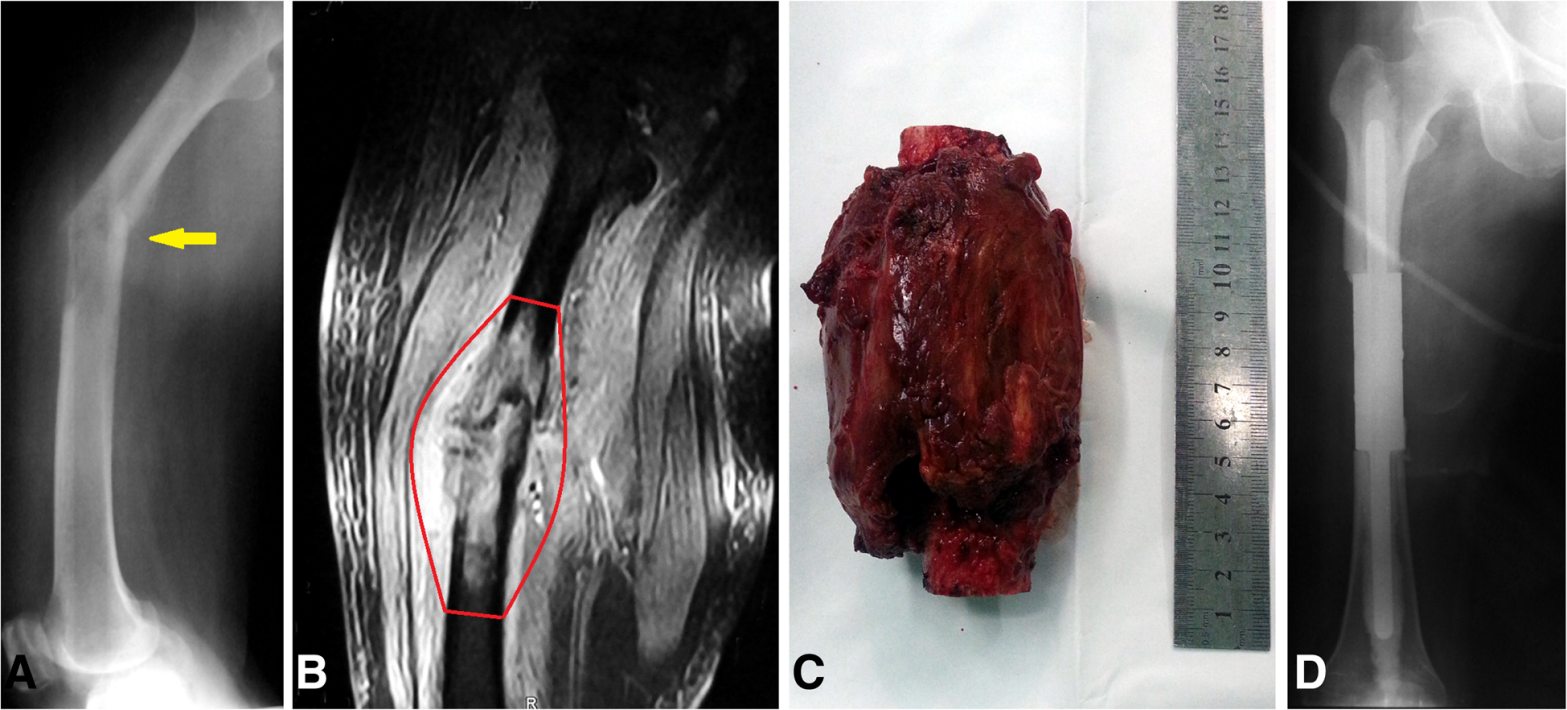
All patients underwent en bloc resection of tumors, followed by reconstruction with second-generation modular intramedullary segmental prostheses characterized by a lap joint. **a** Radiograph of the right femur and (B) coronal magnetic resonance imaging of the upper leg of a 74-year-old woman with rectal carcinoma involving the diaphysis of the femur (Patient 16). The yellow arrow in **a** showing the site of the femoral pathological fracture. The red line in **b** showing the surgical margin. **c** En-bloc resection of a malignant bone tumor. **d** Postoperative radiograph of the femur at 2 months after surgery, showing a stable diaphyseal construction

All patients underwent en bloc resection of tumors, followed by reconstruction with second-generation modular intramedullary segmental prostheses characterized by a lap joint [17] (Fig. 1). All prosthesis intramedullary stems were fixed with bone cement and an assistant plate was used when the intramedullary stem was shorter than 5 cm. The biomechanical analysis showed that the use of assistant plate improved the rigidity of anti-tension and anti-torsion, and diminished the risk of prosthetic loosening and dislocation [18]. All patients in this series were followed-up regularly and underwent postoperative limb function evaluation. The regular follow-up was performed in the outpatient department. Follow-up records included patients’ feeling, proprioception, imaging reexamination, and limb function evaluation, which was quantified by the Musculoskeletal Tumor Society (MSTS) score [19].

### Statistical analysis

All patient analysis was conducted with regard to survivorship, complications, site of complication, and functional outcomes. First, the following descriptive statistics were calculated: frequency, percent, mean and standard deviation. Thereafter, comparisons were performed using Student’s t-test for continuous variables and Pearson’s Chi square test/Fisher’s exact test for categorical variables. The level of statistical significance was set at *p* < 0.05. All statistical analyses were performed using the GraphPad Prism Software (Version 5; GraphPad Software, Inc., La Jolla, CA, USA).

## Results

**Fig. 2:**
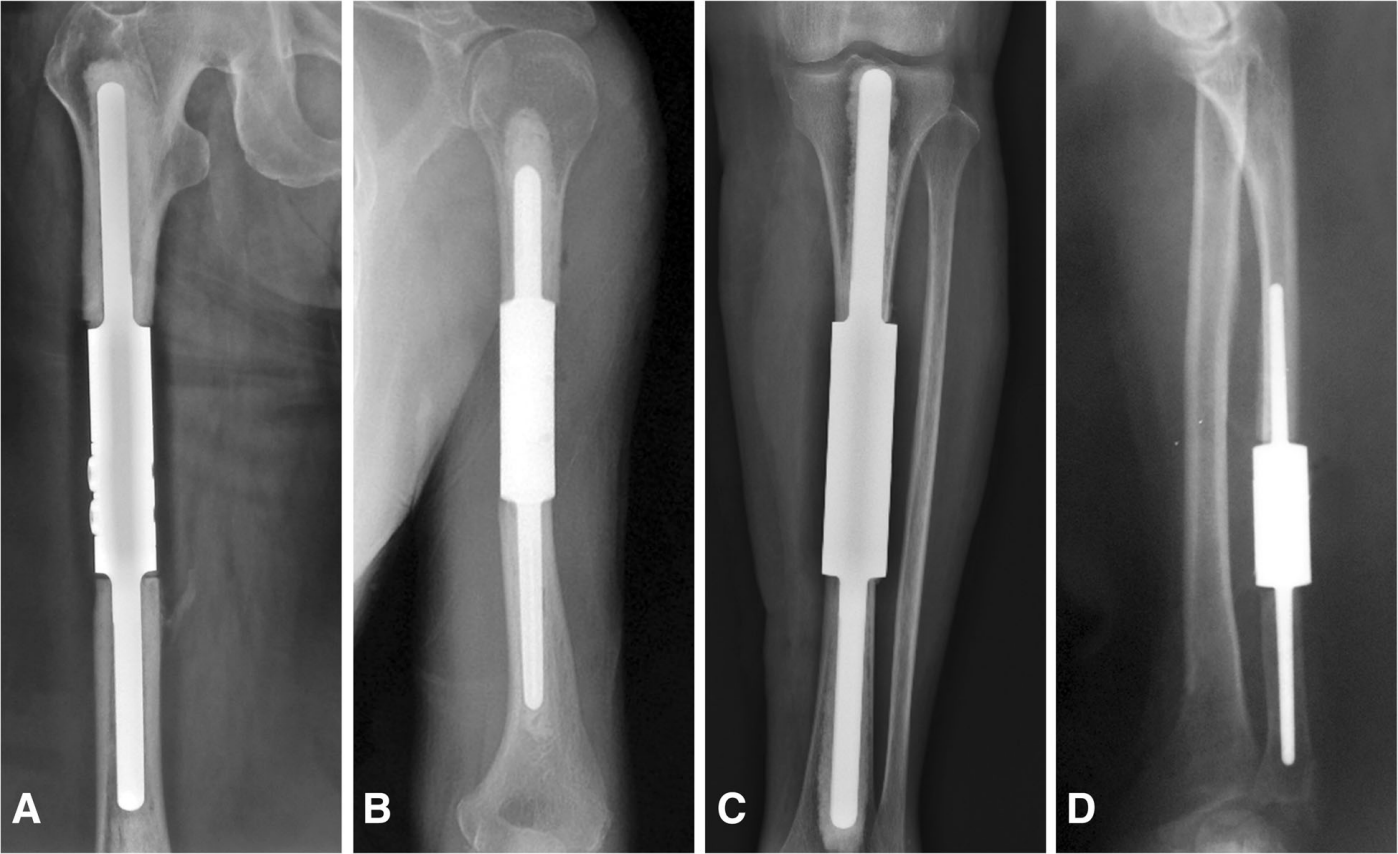
In this study, modular intramedullary segmental prosthesis reconstruction was performed in all patients, which included 30 (61.2%) femora, 13 (26.5%) humeri, five (10.2%) tibiae, and one (2.0%) ulna. Radiographs showing examples of diaphyseal reconstruction of the (**a**) femur, **b** humerus, **c** tibia, and **d** ulna using modular intramedullary segmental prostheses. For these four patients, no assistant plate had been used because the remaining bone marrow cavity was long enough

**Fig. 3:**
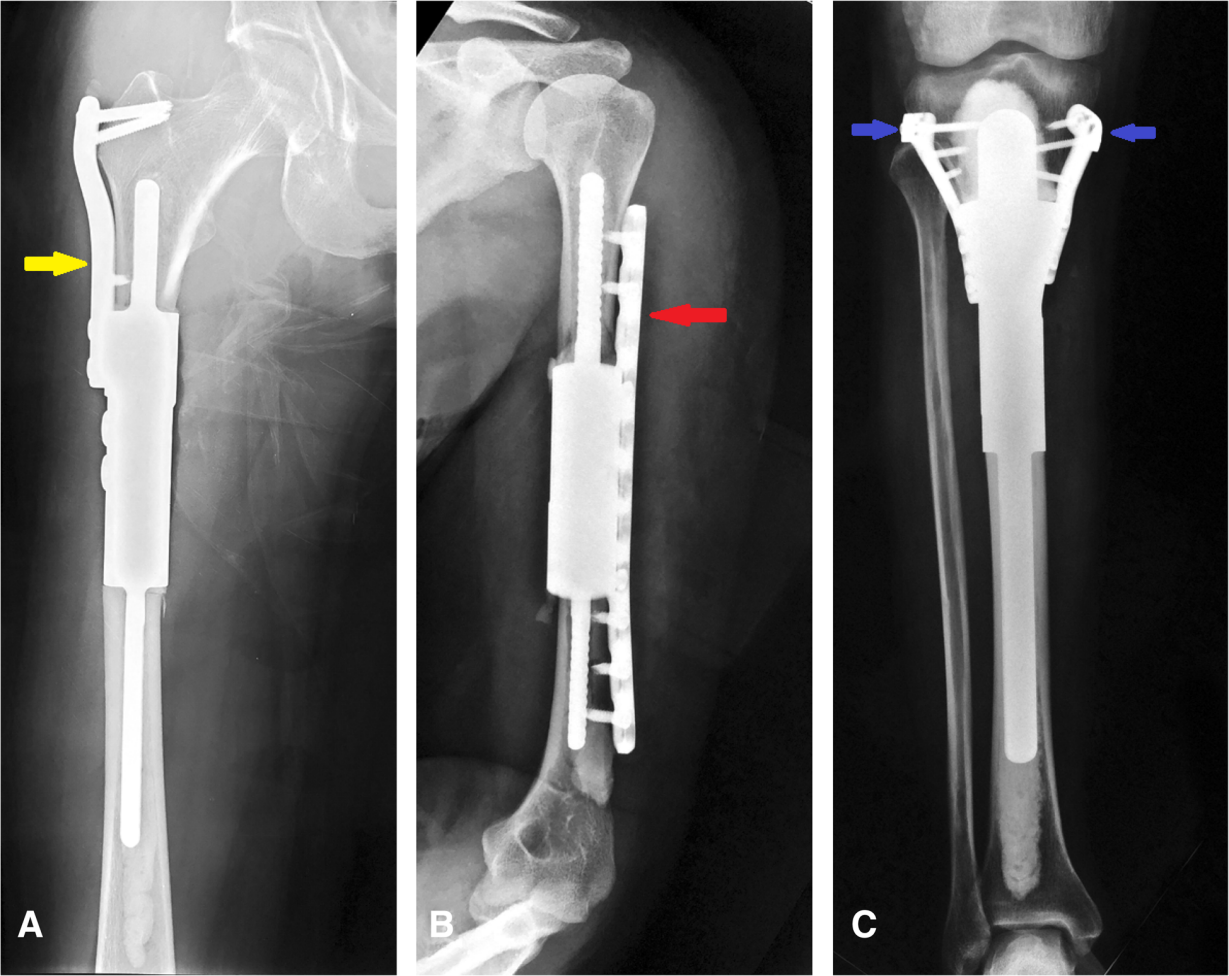
All prosthesis intramedullary stems were fixed with bone cement and an assistant plate was used when the intramedullary stem was shorter than 5 cm. Radiographs evidencing the use of assistant plates in (**a**) the femur and (**c**) tibia. The yellow arrow in **a** showing the unilateral assistant plate used in the proximal femur. The biomechanical analysis showed that the use of assistant plate improved the rigidity of anti-tension and anti-torsion, and diminished the risk of prosthetic loosening and dislocation. The use of an assistant plate in the humerus can effectively reduce the stress and the risk of loosening of the prosthesis. The red arrow in **b** showing a long assistant plate used in humerus. In this study, 12 patients accepted assistant plate fixation of the prothesis, including 5 for the humerus, 6 for the femur, and one for the tibia

There were 49 patients enrolled in the study, including 23 males and 26 females, with a mean age of 63.3 years (range, 13–83 years). Modular intramedullary segmental prosthesis reconstruction was performed in all patients, which included 30 (61.2%) femora, 13 (26.5%) humeri, five (10.2%) tibiae, and one (2.0%) ulna (Fig. 2). Seven patients (14.3%) with a mean age of 48.9 years (range, 13–83 years) had primary tumors and 42 (85.7%) patients with a mean age of 65.7 years (range, 45–82 years) had metastatic lesions. Of the 49 cases, 37 cases had a pathological fracture of the surgical site. The histological diagnosis, patient age, surgical sites, preoperative and surgical details, follow-up, complications, and MSTS scores for each patient are reported in Table 1. The mean defect size was 92.4 mm (humerus = 80.0 cm, tibia = 98.0 mm, femur = 97.3 mm). The mean reconstruction length was 112.9 mm for primary bone tumor and 89.0 mm for diaphyseal metastasis. Twelve patients accepted assistant plate fixation of the prothesis, including 5 for the humerus, 6 for the femur, and one for the tibia (Fig. 3). The average duration of follow-up was 16.0 months (range, 3–38 months) for patients with primary bone tumors and 13.2 months (range, 3–79 months) for those with diaphyseal metastasis. At latest follow-up, 31 (63.3%) of patients died of disease, one patient (2.0%) died of other causes, four (8.2%) showed no evidence of disease, and 13 (26.5%) were alive with disease.

Overall, 11 patients (22.4%) developed complications in this series (Table 2). The complications were divided into four classes, according to the five modes of failure for prosthetics proposed by Henderson et al. [20]. Of these 11 patients, five (10.2%; patients 3, 13, 29, 43, and 26) had type I failure (soft tissue failure), which included radial nerve injury (patients 3 and 13) and delayed incision healing (patients 29, 43, and 26), two cases (4.1%; patients 4 and 11) had type II failure (aseptic loosening), which occurred at the proximal humerus prosthetic stem, one case (2.0%; patient 27) had type III failure (structural failure), which occurred between the prosthetic axis and the biologic axis, and three cases (6.1%; patients 18, 45, 48) had type V failure (tumor progression), which occurred in three of the patients with primary bone tumor. Two of the 13 patients who accepted humeral prosthetic reconstruction presented postoperative radial nerve paralysis. Although nerve rehabilitation therapy was undertaken as soon as possible post-surgery, the clinical effect of nerve function recovery was not obvious. Delayed incision healing occurred in three patients, including two with reconstruction in the thighs and one in the leg, for whom the incisions healed only after incisional debridement and repeated dressing replacements. No periprosthetic infections occurred in this series because of good soft tissue coverage. All patients who accepted prosthetic reconstruction of the tibia got muscle flap transposition in order to protect the prosthesis and reduce the risk of periprosthetic infection. One patient (patient 4) experienced humeral prosthesis aseptic loosening at 7 months, visible on imaging examination, without pain or other clinical symptoms. The patient refused prosthesis revision and had limited upper limb function until she died. Another patient (patient 11) experienced humeral prosthesis aseptic loosening and refused prosthesis revision as well. The difference was that this patient got aseptic loosening at 12-months post-surgery and still lived with acceptable limb function. One patient (patient 27) experienced structural complications which were found on postoperative X-ray examination. An angle of almost 13 degrees was found between the prosthetic force axis and the biological force axis. No clinical symptoms were apparent after the operation. Unfortunately, the patient died of tumor progression at 3 months post-surgery. One patient (patient 18) accepted hip dislocation at 12 months after femoral prosthetic reconstruction because of recurrent osteosarcoma in the femur. One patient (patient 45) accepted thigh amputation at 7 months after tibial prosthetic reconstruction because of Langerhans cell sarcoma recurrence in popliteal. Another patient (patient 48) accepted thigh amputation at 7 months after tibial prosthetic reconstruction because of malignant fibrous histiocytoma recurrence in the leg. The first two patients (patient 18 and 45) lived without tumor recurrence after amputation, while the third patient (patient 48) died because of tumor progression.

**Table 2:** Cases of implant failure categorized according to criteria proposed by Henderson et al

Type of failure	Number of patients (%)
I (soft tissue failure)	5(10.2%)
II (aseptic loosening)	2(4.1%)
III (structural failure)	1(2.0%)
IV (infection)	0(0%)
V (tumor progression)	3(6.1%)

Although the incidence of complications in primary tumor patients (4/7, 57.1%) was higher than that in patients with metastatic tumors (7/42, 16.7%) (*p* = 0.036), the complications seen in the former were mostly local tumor recurrence and incision complications. Aseptic loosening and mechanical complications were not common for patients with resected primary tumors, although the reconstruction length difference was statistically significant (*p* = 0.023). No statistically significant differences were observed in limb function, while the mean MSTS score was 21.2 in femora, 19.6 in humeri, and 17.8 in tibiae (*p* = 0.134).

## Discussion

The optimal reconstruction of diaphyseal defects after tumor resection remains controversial. Vascularized fibula autograft is considered to be the best method for early bone graft healing. However, the average weight-bearing time for reconstruction of femoral shaft defects is 13 months [21]. Allografts are considered to be the best method for the reconstruction of bone defects. However, the infection rate is 10–30% [5, 22], the rate of nonunion is as high as 30–63% [22, 23], and the incidence of bone graft fracture is 14–42% [5, 24–26]. In addition, postoperative radiotherapy and chemotherapy may cause the failure of biological reconstruction and postoperative complications [27]. In contrast to the above methods, the clinical outcome of segmental prosthesis reconstruction is not affected by the length of bone defect. The stable prosthesis can be obtained when the fixed length of the prosthetic stem is higher than 5 cm [13]. Assistant plates can be used to reinforce prosthesis stability if the length of the intramedullary stem is not more than 5 cm. Immediate postoperative stability of the segmental prosthesis is beneficial to the recovery of limb function after operation. In this series, all patients that accepted segmental prosthesis diaphyseal reconstruction after tumor resection experienced pain relief and early limb function recovery. This research attempts to clarify the criteria that deem a patient suitable for this operation. First, patients with both diaphyseal metastases in long bones and pathological fractures should be advised to accept segmental prosthesis. Second, patients with diaphyseal metastases in long bones with high risk of fracture and poor bone condition should also consider accepting segmental prosthesis. Third, segmental prosthesis could be considered as an option in limb salvage surgery for poor prognosis patients with primary malignant tumors in the diaphysis.

Aseptic loosening is the main factor contributing to segmental prosthesis failure, reported to account for 25% in the literature [11]. Clinical studies have found that prosthesis loosening is much more common in the humerus than the femur [9, 11]. In this series, aseptic loosening occurred in two patients with lesions in the humerus. Interestingly, no loosening (0/5) was observed when an assistant plate was used for prosthesis fixation, whereas loosening (2/8) occurred when the prosthesis was fixed with bone cement only. Similar to compressive stress in femoral prosthesis, the tensile and torsional stresses on humeral prosthesis may be the main causes of segmental prosthesis loosening in the humerus. The use of an assistant plate in the humerus can effectively reduce the stress and the risk of loosening of the prosthesis. The incidence of segmental prosthesis loosening in this study was lower than previously reported [10, 11, 17]. The reasons for this have been analyzed and listed. First, most patients in this study had short survival time, with a median of 9 months. Second, assistant plates were used in order to reduce loosening which may be caused by the short intramedullary stem. Third, the average age of these patients was 63.3 years and most presented with multiple metastases which imply less daily activities and, consequently, lower risk of loosening. Fourth, prosthesis stem fixation with polymethyl methacrylate (PMMA) for this intercalary segmental prosthesis is advantageous because of the limited survival time and poor bone condition. Some studies have demonstrated increased postoperative function recovery in patients with cemented stems [10, 28].

The average function result of all 49 patients, as measured by the MSTS score, was 20.6 points, which is comparable to that reported in other studies [10, 11, 17]. Although the rehabilitation of limb function was not complete, patients experienced adequate pain relief and self-care limb functions. In particular, the short postoperative recovery time and early full weight-bearing are particularly valuable for patients with short life expectancy.

It is necessary to alert readers to be aware of the limitations of this study. Firstly, the number of patients is still small although it is the largest series among the reports owing to the fact that prosthesis reconstruction for diaphyseal defects following malignant tumor resection are rare. Without large sample statistical analysis, it is hard to make any definitive statements regarding the differences of recurrences, complications and postoperative function among different tumors and locations. Secondly, this is a multicentric retrospective study and the patients’ treatments were decided by three experienced bone tumor surgeon teams respectively, consequently, the differences among surgical technologies can-not be avoided. Nevertheless consensuses of treatments had been made by these surgeon teams. Thirdly, the mean follow-up is short and additional clinical events might occur with longer follow-up. Nevertheless, 32 patients in all 49 patients were deceased in this follow-up.

## Conclusions

In conclusion, segmental prostheses represent an optional method for diaphyseal defect reconstruction after bone metastasis resection. The main advantages of this approach include easy operation, early weight-bearing and acceptable incidence rate of complications. Moreover, segmental prostheses in the humerus experienced more complications than those used to treat lesions in the femur.

## Data Availability

The datasets used and/or analysed during the current study are available from the corresponding author on reasonable request.

## Abbreviations

CT: Computer tomography
ECT: Emission Computed Tomography
MR: Magnetic resonance
MSTS: Musculoskeletal Tumor Society
PMMA: Polymethyl methacrylate
